# Neolithic expansion and the 17q21.31 inversion in Iberia: an evolutionary approach to H2 haplotype distribution in the Near East and Europe

**DOI:** 10.1007/s00438-022-01969-0

**Published:** 2022-11-10

**Authors:** Ibone Espinosa, Miguel A. Alfonso-Sánchez, Luis Gómez-Pérez, Jose A. Peña

**Affiliations:** Departamento de Genética, Antropología Física y Fisiología Animal, Universidad del País Vasco (UPV/EHU), Apartado 644, 48080 Bilbao, Spain

**Keywords:** MAPT, Tau protein, Basques, Founder effect, Genetic drift, Most recent common ancestor

## Abstract

The chromosomal region 17q21.31 harbors a 900 kb inversion polymorphism named after the microtubule-associated protein tau (MAPT) gene. Since no recombination occurs, two haplotypes are recognized: a directly oriented variant (H1) and an inverted variant (H2). The H2 haplotype features a distribution pattern with high frequencies in the Near East and Europe, medium levels in South Asia and North Africa, and low levels elsewhere. Studies of this genomic region are relevant owing to its likely association with numerous neurodegenerative diseases. However, the causes underlying the geographic distribution of the haplotype frequencies remain a bone of contention among researchers. With this work, we have intended to outline a plausible hypothesis on the origin of the high European H2 frequencies. To that end, we have analyzed an extensive population database (including three new Iberian populations) to explore potential clinal variations of H2 frequencies. We found a sigmoidal frequency cline with an upward trend from South Asia to Europe. The maximum value was detected in the Basques from Gipuzkoa province (0.494) with the curve’s inflection point in the Near East. From our results, we suggest that the most likely scenario for high H2 frequencies in Europe would be a founding event in the Near East during the late Paleolithic or early Neolithic. Subsequently, such H2 overrepresentation would have reached Europe with the arrival of the first Neolithic farmers. The current frequencies and geographic distribution of the 17q21.31 inversion suggest that the founding events mainly affected the H2D subhaplotype.

**Supplementary Information:**

The online version contains supplementary material available at 10.1007/s00438-022-01969-0.

## Introduction

The chromosomal region 17q21.31 harbors a 900 kb inversion polymorphism (Stefansson et al. 2005), named after one of the most studied genes in this region because of its plausible association with several neurodegenerative diseases (Ballatore et al. 2007; Arendt et al. 2016): the microtubule-associated tau protein (MAPT) gene. Linkage disequilibrium resulting from changes in the orientation of the 17q21.31 genomic region has led to define two distinct haplotypes: H1 considered the common, directly oriented variant, and the inverted H2 variant (Donnelly et al. 2010). In addition, some authors have identified detectable levels of MAPT haplotype diversity, thus distinguishing different subhaplotypes such as H2' and H2D. Among them, H2' would be the ancestral H2 haplotype and H2D, carrying some duplications, would be the derived haplotype (Steinberg et al. 2012). The H1 haplotype is globally widespread, being present in all human populations studied to date (Evans et al. 2004). On the other hand, the less frequent H2 haplotype is virtually absent in sub-Saharan Africa, East Asia, the Americas, and Oceania, thus contrasting with the notable frequencies observed in Southwest Asia, Europe and North Africa (Evans et al. 2004; Donnelly et al. 2010; Steinberg et al. 2012; Alves et al. 2015).

Different estimates of the time to the Most Recent Common Ancestor (MRCA) of the inversion have been obtained so far, leading to divergent hypotheses on the origin of the H2 ancestral haplotype. These estimates range from 3 million years (Stefansson et al. 2005) to less than 100 kyrs (Donnelly et al. 2010). The source area is also controversial, as these authors suggest that it could be either Africa (Stefansson et al. 2005; Donnelly et al. 2010) or southwest Asia (Donnelly et al. 2010). However, demographic events that might have originated the current worldwide distribution of the H2 haplotype remain relatively unexplored. Intending to provide new evidence on why H2 is especially frequent in southern Europe, herein we analyzed an extensive population database (including three new Iberian populations) to look for potential clinal variations of H2 frequencies across the territory involved.

## Materials and methods

### Study samples

Aimed at obtaining representative samples of the genetic variability in the Iberian Peninsula, our study included three population samples from two geographically and genetically differentiated Spanish regions. Thus, we assessed the genetic diversity of the Cantabrian watershed by sampling two Basque areas. On the other hand, analysis of the Mediterranean region relied on a sample from the Valencian community.

A recent study suggested that Basque human groups might have undergone remarkable population isolation since, at least, the Iron Age. Logically, this fact would have been crucial to make autochthonous Basques an anthropologically distinct population (Olalde et al. 2019). Besides the complex orography of the Basque area, several studies have claimed that Euskera, the native Basque language of pre-indo-European origin, could have represented a linguistic barrier to gene flow with neighbouring populations speaking a language other than Euskera (Alfonso-Sánchez et al. 2005; García-Obregón et al. 2007). Thus, we gathered samples from two distinct zones of the traditional Basque area essentially distinguished by the prevalence of Euskera, a factor frequently neglected in investigations involving Basques. Specifically, study samples stemmed from different regions of the Gipuzkoa province and the Baztan valley, located in the northern end of Navarre province. At present, Euskera persists in both areas. Yet, while Gipuzkoa borders other Basque-speaking populations in Spain and the French Basque region (Iparralde), Navarra is mainly surrounded by Spanish territories speaking an Indo-European language (Castilian). Accordingly, Navarre province has traditionally been more permeable to gene flow from outside the Basque-speaking area (Pérez-Miranda et al. 2005).

As previously mentioned, we examined the genetic heterogeneity of the Mediterranean basin by analyzing a sample from Valencia. Contrarily to the population isolation of the Basque area, Valencia seems to have been under the influence of recurrent gene flow events since the Iron Age, coming from both the Central and Eastern Mediterranean and North Africa. (García‐Obregón et al. 2006; Olalde et al. 2019).

### MAPT haplotyping

Assignment of MAPT haplotypes/subhaplotypes involved typing of two single nucleotide polymorphisms (SNPs), namely rs10514879 and rs199451 (see Supplementary Table 1), using the high-resolution melting (HRM) assay (Alfonso-Sánchez et al. 2018).

To refine the MAPT haplotype identification, we further examined four short tandem repeats (STRs) (*MAPT07*, *08*, *09* and *14*) located within the boundaries of the inversion of the MAPT gene, following the protocol developed by Donnelly et al. (2010). Microsatellites were typed via PCR amplification with fluorescently labeled primers, the lengths of the PCR products determined on an ABI Prism 310 Genetic Analyzer (Applied Biosystems, Foster City, CA).

### Statistical and phylogenetic analysis

MAPT haplotypic frequencies in European, Near Eastern and South Asian human samples were collected from published studies to broaden the geographical context of this study (Supplementary Table 2). As mentioned, these geographic regions show higher MAPT*H2 haplotype frequencies than those reported for other continents (Evans et al. 2004). Alves et al. (2015) also referred to relatively high H2 frequencies in North Africa, but no published frequencies were found in the relevant literature. In the next step, H2 frequency data were used to assess genetic heterogeneity in selected population clusters by a hierarchical analysis of molecular variance (AMOVA) using the program Arlequin v3.5 (Excoffier and Lischer 2010). We further explored potential geographic (spatial) patterns of haplotype frequencies using the GenoCline software (Peña et al. 2021), which can detect frequency gradients either in the form of linear or sigmoid genetic clines.

Phylogenetic relationships were examined by constructing a genealogy of STR/SNPs haplotypes using the median-joining network approach (Bandelt et al. 1995) in the Network v10.2 software (Fluxus Technology). Subsequently, STR/SNPs haplotype data were input to calculate the time elapsed to the most recent common ancestor (MRCA) of the Iberian haplotypes, following the method of Stephens et al. (1998). To estimate the number of generations to the MRCA, we considered a mutation rate between 0.0005 and 0.0010, as per Donnelly et al. (2010). We also assumed a recombination rate of zero, considering that all four STRs examined are within the inversion. Finally, we set an average generation length of 25 years.

For the sake of gaining insights into the evolution of variations in MAPT haplotype frequencies over time, we identified and compiled MAPT haplotypes from ancient DNA databases for Europe (Lazaridis et al. 2014, 2016, 2017; Mathieson et al. 2015, 2018; Fu et al. 2016; Lipson et al. 2017; Olalde et al. 2018, 2019) and South Asian (Narasimhan et al. 2019) samples.

Finally, H2' and H2D frequencies were compiled for different continents (Supplementary Table 3), aiming to elucidate which of these two subhaplotypes could account for the origin of the high MAPT*H2 European frequencies.

## Results

Table 1 presents MAPT haplotype and subhaplotype frequencies for Iberian samples. In Gipuzkoa Basques, tau H1 and H2 haplotype frequencies were similar, around 50% (H1: 50.6%, H2: 49.4%). In contrast, the haplotype frequencies of the Navarrese Basques and Valencians were in line with European populations, with a clear predominance of H1 (68.9% and 74.2%, respectively). In Gipuzkoa, the high H2 frequency is primarily due to the high incidence of the inverted haplotype with duplication (H2D: 98.8% of total H2). Interesting results emerged by applying the likelihood ratio test (or *G* test) to evaluate population differentiation. Thus, we found statistically significant differences for H2 haplotype frequencies between the pool of Basque collections and the Valencian sample (*G* = 10.16, *df* = 1, *P* < 0.01). This genetic heterogeneity was determined by Gipuzkoa Basques. In this way, while H2 frequencies between Navarrese Basques and Valencians did not show significant differences (*G* = 1.26, *df* = 1, *P* = 0.262), we found statistically significant differences between Gipuzkoan and Navarrese Basques (*G* = 13.87, *df* = 1, *P* < 0.001).

**Table 1:** Frequency estimates (± standard error) of direct (H1) and inverted (H2) MAPT haplotypes in three Iberian populations

	Gipuzkoa	Navarra	Valencia
*N*	180	210	182
H1	0.506 ± 0.037	0.689 ± 0.035	0.742 ± 0.032
H2	0.494 ± 0.037	0.311 ± 0.035	0.258 ± 0.032
H2'	0.006 ± 0.006	0.033 ± 0.013	0.022 ± 0.011
H2D	0.488 ± 0.037	0.278 ± 0.033	0.236 ± 0.031

Also displayed are the frequencies of inverted subhaplotypes without duplication (H2'), and with duplication (H2D)

*N* sample size as chromosome number

**Fig. 1:**
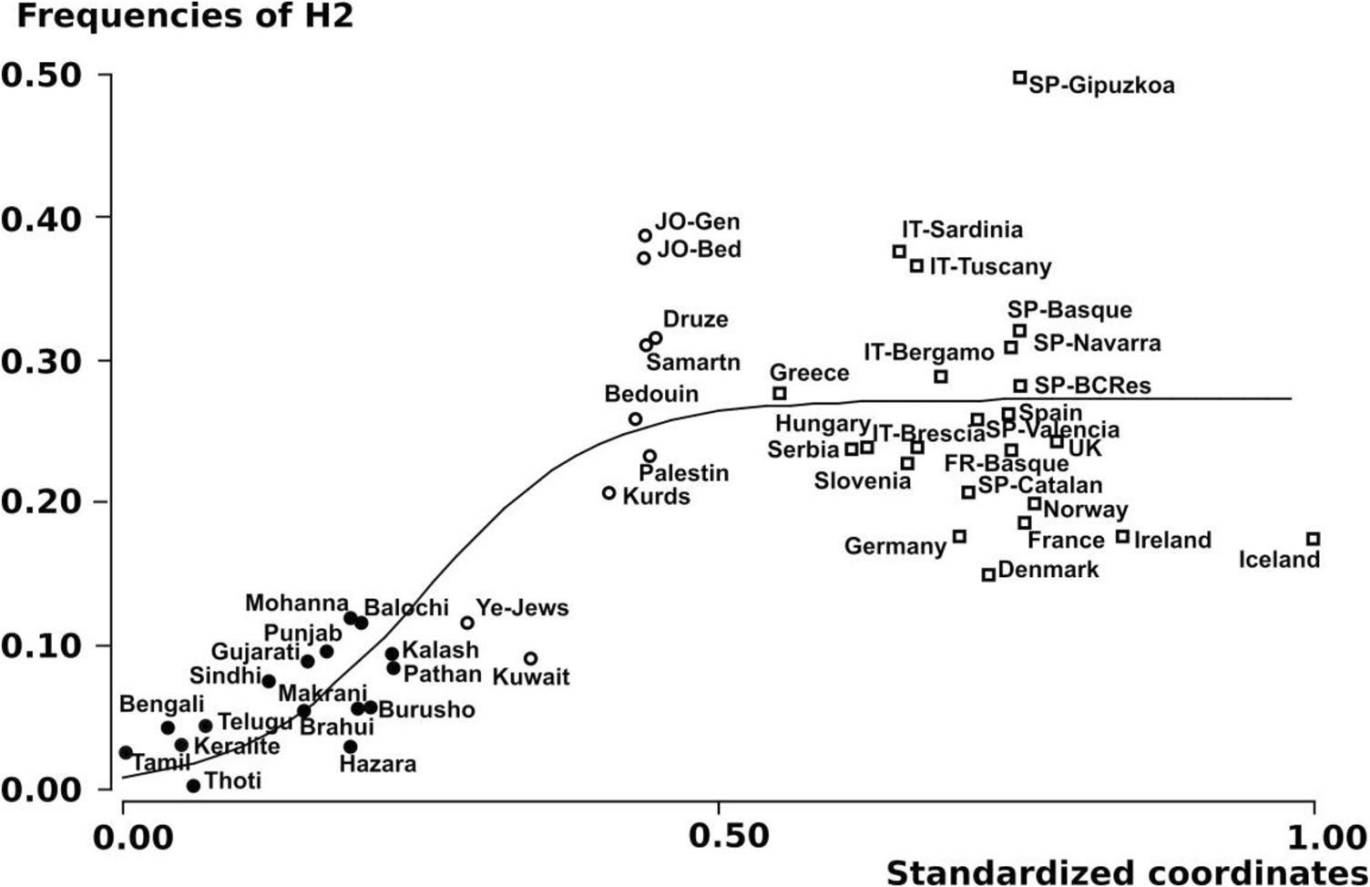
Regression of MAPT*H2 haplotype frequencies on geographic coordinates in a set of South Asian and European populations. The *y*-axis is a coordinate axis with a rotation of 312º relative to the North. Results matched the sigmoid function $y=\frac{a}{\left(1+{e}^{\left(-b*\left(x-c\right)\right)}\right)}$, with *a* = 0.2687, *b* = 13.8090 and *c* = 0.2519. For population labels see Supplementary Table 2. Solid circles represent South Asia, open circles the Near East, and squares Europe

There was a distinctive pattern in the MAPT*H2 haplotype frequency distribution throughout the territory from Europe to South Asia (see Fig. 1 and Supplementary Table 2). Haplotype frequencies tend to be lower in South Asia and the eastern part of the Near East, ranging from 0.00 to 0.13. In contrast, in the western part of the Near East and Europe, H2 frequencies tend to be much higher, ranging from 0.15 to 0.49.

An analysis of the spatial structuring of haplotype frequencies using the GenoCline program revealed a sigmoid cline with a Southeast-Northwest orientation, specifically with an azimuth of 312° to the North. All sigmoid function parameters were statistically significant, including the ordinate scaling factor (*P* < 10^–6^), slope (*P* < 10^–3^), midpoint (*P* < 10^–6^), and F ratio (*P* < 10^–6^).

The midpoint of the threshold (i.e., the curve’s inflection point) appeared around the Near East, indicating a shift in the function's trend in this region. As a result, populations from the eastern portion of the Near East showed frequencies below 0.15 (Kuwait: 0.09 and Yemenite Jews: 0.12), while those in the western area featured frequencies above this value. (Jordan: 0.39, Bedouin of Jordan: 0.37, Druze: 0.32, Samaritan: 0.31, Bedouin: 0.26, Palestine: 0.23, and Kurds: 0.21).

**Fig. 2:**
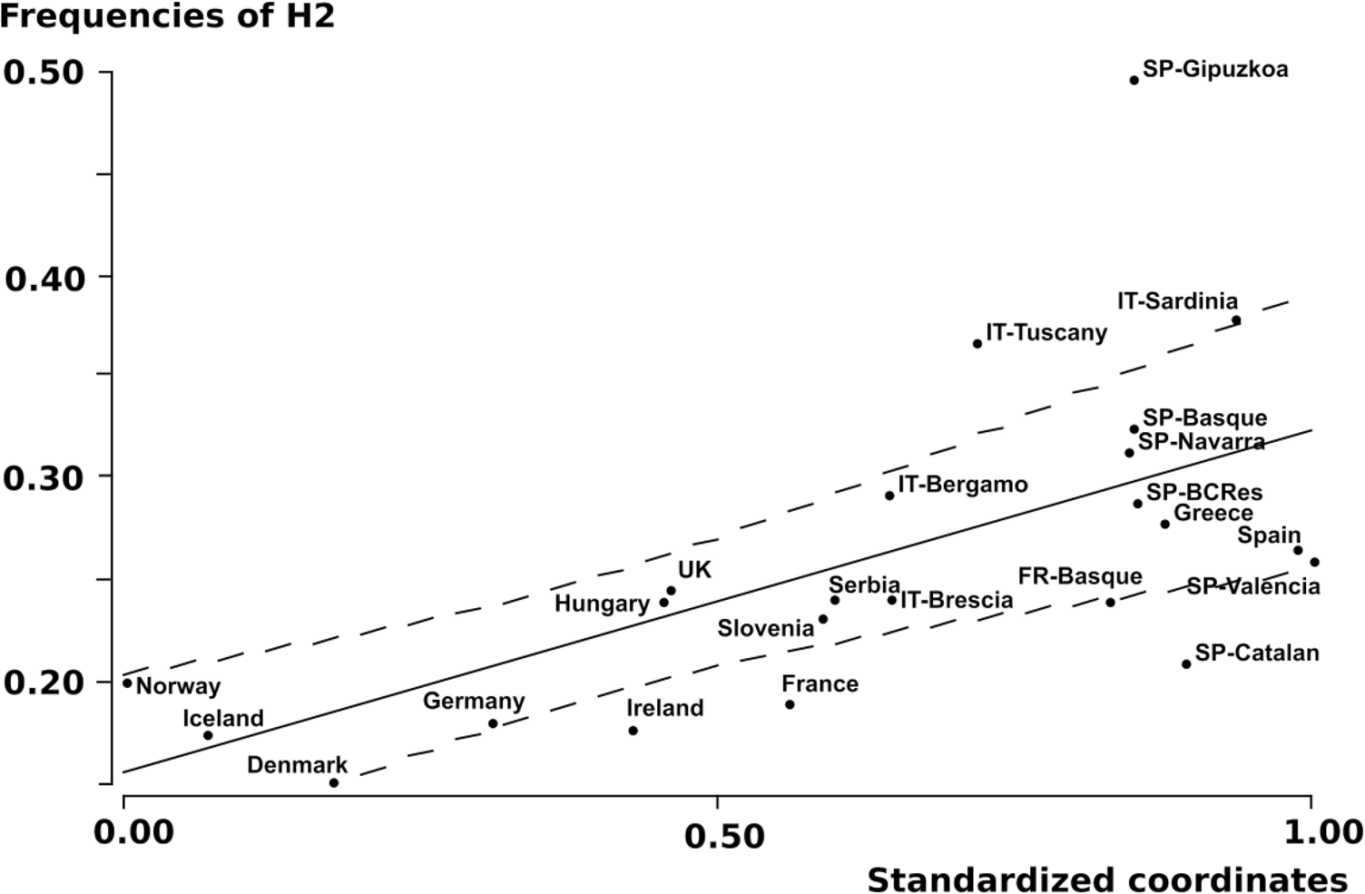
Linear regression of MAPT*H2 haplotype frequencies in European populations against a rotating coordinate axis. Parameter values match the function *y* = *a* + b*x, where *a* = 0.1553, and *b* = 0.1634. The axis is rotated 190º relative to the North. Dashed lines are 95% confidence limits

Through AMOVA, we compared the H2 frequencies between the geographic regions delimited by sigmoid regression (South Asia, including Eastern-Near East versus Europe, including Western-Near East). All of the fixation indices obtained were highly significant at *P* < 10^–5^ (F_ST_: 0.1455, F_SC_: 0.0025 and F_CT_: 0.01689), thus confirming genetic heterogeneity between Europe and South Asia in terms of MAPT*H2 frequencies. Further analysis based only on European populations revealed a significant cline for MAPT*H2 (*R*: 0.6094; *P* < 0.01), but in this case, spatial variation in H2 frequencies matched the linear regression model (Fig. 2). The resulting genetic cline was North–South oriented, with an azimuth of 190° relative to the North.

Southern European samples, especially Spanish groups, exhibited high levels of genetic heterogeneity. Spanish populations evidenced a wide range of H2 haplotype frequencies, varying from 0.21 in Catalonia to 0.49 in Gipuzkoa. Overall, such a wide variability could be attributable to the differences between autochthonous Basques and the remaining Spanish samples. In this way, MAPT*H2 frequencies in the non-Basque collections ranged from 0.21 to 0.26, whereas the Basque collections exhibited figures between 0.31 and 0.49. Non-native Basques (residing population of the Basque Country), with a high admixture level between both groups, recorded an intermediate frequency (0.28). Earlier studies have suggested that the French-Basque population has traditionally experienced substantial gene flow from neighbouring non-Basque communities (Calderón et al. 1998, 2000), as reflected by the lower H2 frequency relative to Iberian Basques. The strikingly high frequency of MAPT*H2 in Gipuzkoa (0.49) is noteworthy. Such value represents a maximum among the world populations analyzed to date.

**Fig. 3:**
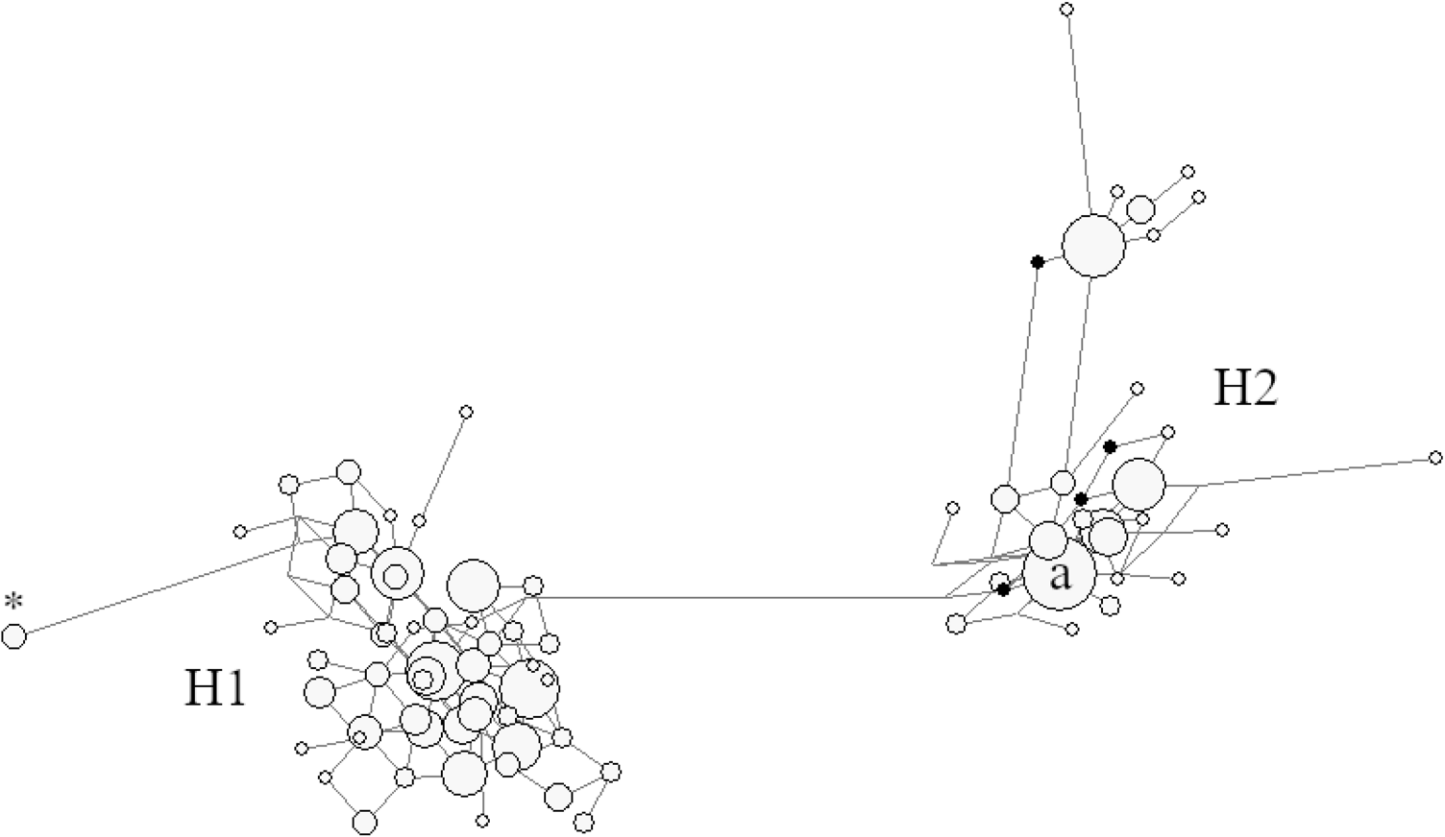
Haplotype phylogenetic network based on four STRs and two SNPs from the MAPT region for several Spanish populations. The left-side group of haplotypes means individuals carrying MAPT*H1 (exception marked with an asterisk), while the right-side clusters correspond to MAPT*H2 individuals. The label a indicates the most frequent H2 haplotype. Solid circles represent H2′ subhaplotypes

To delve into the causes of the high frequency of H2 in Europe, we analyzed four STRs from the MAPT region (Donnelly et al. 2010) in both the study samples (Navarra, Gipuzkoa, and Valencia) and the general population in the Basque Country (Alfonso-Sánchez et al. 2018). Network analysis of the haplotypes generated for this sample set (Fig. 3) depicts one group of haplotypes associated with H1 (left side of the graph) and two groups of haplotypes associated with H2 (right side). Haplotype *a* is the most frequent one (0.075) and presumably the ancestor of all Iberian H2 haplotypes. For this reason, we based on data for haplotype *a* to perform MRCA estimates.

Estimates of the time to the MRCA of Iberian H2 haplotypes ranged between 648 and 1295 generations, i.e. between 16,189 and 32,378 years. The range central value was about 24 kiloyears (kyrs), that is, around the beginning of the Last Glacial Maximum.

A set of previous studies provide genome-wide data on ancient European populations (references in the Methods section). A molecular polymorphism commonly examined in those studies based on ancient DNA from human remains is the SNP rs10514879, a diagnostic marker for MAPT haplotype assignment. Unfortunately, Iberian samples with identifiable genotypes proved rare, then all European individuals pooled. Table 2 shows the frequencies arranged according to different cultural periods. All Paleolithic and Mesolithic samples showed H2 frequencies below the values of extant Europeans. By contrast, frequencies corresponding to the Neolithic, Chalcolithic and Bronze Age fell within the range of present European frequencies, above 0.15.

**Table 2:** Frequency estimates (± standard error) for the MAPT*H2 haplotype in ancient DNA samples from the European continent, arranged by cultural periods

	*N*	H2
Paleolithic & Mesolithic	69	0.043 ± 0.012
Neolithic	109	0.220 ± 0.020
Chalcolithic	65	0.154 ± 0.022
Bronze age	124	0.161 ± 0.017

*N* sample size as chromosome number

**Table 3:** Frequency estimates (± standard error) for the MAPT*H2 haplotype in ancient DNA samples from Central and South Asia, arranged by cultural periods

	*N*	H2
Neolithic	11	0.000 ± 0.000
Chalcolithic	10	0.200 ± 0.063
Bronze age	92	0.228 ± 0.022
Iron age	31	0.065 ± 0.022

*N* sample size as chromosome number

MAPT haplotype assignment was also feasible on ancient Central and South Asian populations from data published by Narasimhan et al. (2019). The number of human remains with identifiable MAPT haplotypes was low, except for the Bronze Age, where 21 out of 92 total chromosomes could be classed as H2 haplotypes. H2 frequency in this group was strikingly high (0.23), a value clearly above frequencies currently observed in Central and South Asia (Table 3).

Unlike African populations, where H2D is generally less frequent than H2' (Steinberg et al. 2012), H2D predominates in Europe, with clearly higher frequencies than H2'. Average H2' frequencies tend to be low, with figures below 0.03 in all continents (East Asia: 0.00; South Asia: 0.02; Africa: 0.02; Europe: 0.03). Alternatively, H2D tend to show more uneven values, with low average frequencies in East Asia (0.00) and Africa (0.01), intermediate in South Asia (0.06), and substantially higher in Europe (0.24), where figures oscillated from 0.09 to 0.49 (see Supplementary Table 3).

## Discussion

Estimates of the time to the MRCA for tau H2 haplotypes in previous works have been far from consistent, ranging from 3 million years (Stefansson et al. 2005) to less than 100 kyrs (Donnelly et al. 2010). The hypothetical area of origin also remains ambiguous and the authors cited above suggest that it could be either Africa (Stefansson et al. 2005; Donnelly et al. 2010) or Southwest Asia (Donnelly et al. 2010). Also, some authors have postulated a selective advantage of H2 (Stefansson et al. 2005; Alves et al. 2015). In any case, the differentiating factor for the tau H2 haplotype among human populations is its uneven worldwide distribution pattern, characterized by high frequencies in the Near East and Europe, medium frequency levels in South Asia and North Africa, and low levels elsewhere (Evans et al. 2004; Donnelly et al. 2010; Alves et al. 2015).

Our study firstly examined the spatial distribution of MAPT haplotype frequencies in the Iberian Peninsula based on three geographically and demographically distinct populations. As one of our main findings, the remarkable high H2 frequencies recorded for the Basque population of Gipuzkoa (0.49) stands out as the highest among all human groups analyzed to date. Gipuzkoa Basques are exceptional because their H2 frequencies are practically equal to H1 (0.51). As a result, we could detect significant genetic heterogeneity between a pool of the Basque samples (Gipuzkoa and Navarre) and the Mediterranean collection (Valencia), on the one hand, and also between the two native Basque groups, most likely due to the unusually high H2 frequency in Gipuzkoa. There has been plenty of evidence that autochthonous Basques from Gipuzkoa are unique in terms of language (predominance of Euskera in rural villages), topography, inbreeding patterns and consanguinity structures, among other traits, relative to other neighboring Basque groups (Alfonso-Sánchez et al. 2005; Pérez-Miranda et al. 2005).

Second, we analyzed the H2 clinal variation in South Asia and Europe, finding a statistically significant sigmoidal cline. Usually, such clines indicate hybrid zones, i.e., contact zones between two genetically divergent populations (Barton and Hewitt 1985). Here, the distinct populations would be the western Near East and Europe, on the one hand, and the eastern Near East and southern Asia, on the other. The hybrid zone would therefore be the Near East.

Then, we utilized a set of four STRs and two SNPs to estimate the time to the MRCA of Iberian H2 haplotypes. Along these lines, including the MAPT haplotypes identified from ancient DNA analysis facilitated the tracking of H2 frequencies across Europe and South Asia over time, especially with the stratification of individuals according to cultural periods. The findings of such an approach supported our dating estimates and the likely origins of the 17q21.31 inversion in Europe.

Based on our estimates, the origin of Iberian H2 haplotypes would have occurred between 16 and 32 thousand years ago, with a central value of 24 thousand years, i.e. around the onset of the Last Glacial Maximum. Therefore, while the MRCA age would be centered around the dawn of the Last Glacial Maximum, the estimate’s upper limit would fall within such an evolutionary milestone. This range of dating allows us to conjecture two plausible evolutionary scenarios. A first look would focus on the frequency peak found in Basques (Gipuzkoa sample). Thus, we could postulate the impact of genetic drift by cumulative effects of founder events during the Last Glacial Maximum in the Franco-Cantabrian refugium and a subsequent spreading associated with the postglacial recolonization of central and northern Europe (Torroni et al. 1998). However, this hypothesis would not explain the high frequencies found in other populations from southern Europe and the Near East. A second scenario would assume founder effect episodes occurred in the Near East during the late Paleolithic or early Neolithic (Alkaraki et al. 2021), with a subsequent dispersal across Europe associated with Neolithic demic diffusion (Harris 2017; Isern et al. 2017). In this case, the high H2 frequency in Gipuzkoa might be explained by more recent genetic drift events associated with the persistent isolation of autochthonous Basque communities until relatively recent times (Alfonso-Sánchez et al. 2008; Olalde et al. 2019). Our findings based on ancient genomes revealed that high H2 frequencies in Europe seem to have emerged during Neolithic times, as Paleolithic and Mesolithic individuals showed relatively low frequencies: only three individuals out of 69 carried the H2 haplotype in Europe.

The Neolithic hypothesis could explain the high H2 levels in Southern Europe, particularly in Sardinia (0.375), whose population gene pool still shares substantial similarities with the early European farming groups (Lazaridis et al. 2014). On the other hand, high frequencies identified in the western region of the Near East might well be genetic traces of the haplotype's origins. The relatively high H2 frequencies in South Asia might mirror the expansion of Eurasian steppe peoples during the Bronze Age (Narasimhan et al. 2019). Finally, comparatively high frequencies in North Africa (Alves et al. 2015) could be the consequence of the Arab migrations stemming from the Near East since the 8th century.

Finally, when considering frequencies of H2' and H2D subhaplotypes, we found that H2', proposed as an ancestral subhaplotype of MAPT*H2 (Steinberg et al. 2012), presented low frequencies throughout all continents. Likewise, H2D, derived from H2' and carrying some duplications, featured the highest frequencies in Europe and intermediate values in South Asia. These values match the current geographic distribution of the tau H2 haplotype. Thus, our findings suggest that genetic drift caused by founder effects in the Near East—which might have contributed to the high H2 frequencies observed there and in Europe—would have primarily affected chromosomes carrying duplications, i.e., H2D.

In summary, the most plausible hypothesis for the origin of high MAPT*H2 frequencies in Europe seems to point to genetic drift by founder events during the late Paleolithic or early Neolithic in the western Near East. According to currently available H2 frequency data and the geographic distribution of chromosomal variants carrying the 17q21.31 inversion, the founder effects would have mainly affected the H2D subhaplotype. H2 overrepresentation would then have entered Europe with the first Neolithic farming communities, on the one hand, and spread to South Asia with the migrations and expansions of Indo-European peoples. The robustness and scope of our findings could be improved by increasing the number of ancient DNA samples from different cultural periods in Europe, North Africa, and Southwest Asia to refine both the timing and the hypothetical dispersal routes of H2 haplotypes.

## Supplementary Information

Below is the link to the electronic supplementary material.

Supplementary file1 (PDF 84 KB)
