# Supplementary material for: Neolithic expansion and the 17q21.31 inversion in Iberia: an evolutionary approach to H2 haplotype distribution in the Near East and Europe
Source: Mol Genet Genomics. 2022 Nov 10;298(1):153–60. doi: 10.1007/s00438-022-01969-0 (PMC9816301; doi:10.1007/s00438-022-01969-0)
Supplement: Supplementary file 1 — Supplementary file1 (PDF 84 KB) [file 438_2022_1969_MOESM1_ESM.pdf]

SUPPLEMENTARY MATERIAL

Supplementary Table 1. Assignment of MAPT haplotypes and subhaplotypes according to SNPs rs10514879 and rs199451.

SNP	rs10514879 allele	rs199451 allele
H1	G	G
H2	A	G/A
H2'	A	G
H2D	A	A
Source	Donnelly et al. (2010)	Steinberg et al. (2012)

Supplementary Table 2. Populations included in the analyses, population labels, sample sizes, haplotype H2 frequencies, and references

Population	Region	Label	2N*	H2	Reference
**Gipuzkoa**	Europe	SP-Gipuzkoa	180	0.494	Present study
**Navarra**	Europe	SP-Navarra	210	0.311	"
**Valencia**	Europe	SP-Valencia	182	0.258	"
Basque Country residents	Europe	SP-BCRes	46	0.283	Alfonso-Sánchez et al. (2018)
Bergamo Italians	Europe	IT-Bergm	58	0.290	Evans et al. (2004)
Brescia Italians	Europe	IT-Bresc	100	0.240	Ghidoni et al. (2006)
British	Europe	UK	126	0.245	1000G
Catalonia Spanish	Europe	SP-Catalan	84	0.209	Donnelly et al.(2010)
Denmark	Europe	Denmark	102	0.150	"

France	Europe	France	58	0.189	"
French Basques	Europe	FR-Basque	48	0.239	"
Germany	Europe	Germany	512	0.180	Winkler et al. (2007)
Greece	Europe	Greece	112	0.277	Donnelly et al. (2010)
Hungary	Europe	Hungary	174	0.239	"
Iceland	Europe	Iceland	3760	0.175	Stefansson et al.(2005)
Ireland	Europe	Ireland	236	0.177	Donnelly et al. (2010)
Norway (Trondheim)	Europe	Norway	882	0.200	Skipper et al. (2004)
Sardinia Italians	Europe	IT-Sardn	70	0.375	Donnelly et al. (2010)
Serbia	Europe	Serbia	382	0.240	Winkler et al. (2007)
Slovenia	Europe	Slovenia	52	0.231	ALFRED
Spain	Europe	Spain	212	0.264	1000G
Spanish Basques	Europe	SP-Basque	152	0.322	Donnelly et al. (2010)
Tuscany Italians	Europe	IT-Tuscn	180	0.364	1000G
Bedouins	Middle East	Bedouin	90	0.260	Jakobsson et al. (2008)
Bedouins of Jordan	Middle East	JO-Bed	300	0.370	Alkaraki et al. (2021)
Druze	Middle East	Druze	190	0.316	Donnelly et al. (2010)
Jordans	Middle East	JO-Gen	594	0.386	Alkaraki et al. (2021)
Iraqi Kurds	Middle East	Kurds	48	0.208	ALFRED
Kuwait	Middle East	Kuwait	32	0.094	Donnelly et al. (2010)
Palestinians	Middle East	Palestin	102	0.235	"
Samaritans	Middle East	Samartn	82	0.310	"
Yemenite Jews	Middle East	Ye-Jews	86	0.119	"
Balochi	South Asia	Balochi	50	0.120	"
Bengalí	South Asia	Bengalí	170	0.047	1000G
Brahui	South Asia	Brahui	50	0.060	Donnelly et al. (2010)
Burusho	South Asia	Burusho	50	0.060	"

Gujarati	South Asia	Gujarati	210	0.091	1000G
Hazara	South Asia	Hazara	64	0.033	Donnelly et al. (2010)
Kalash	South Asia	Kalash	50	0.100	"
Keralite	South Asia	Keralite	60	0.033	"
Mohanna	South Asia	Mohanna	108	0.122	"
Negroid Makrani	South Asia	Makrani	56	0.056	"
Pathan	South Asia	Pathan	46	0.087	"
Punjabi	South Asia	Punjabi	192	0.099	1000G
Sindhi	South Asia	Sindhi	50	0.080	Donnelly et al. (2010)
Tamil	South Asia	Tamil	206	0.029	1000G
Telugu	South Asia	Telugu	206	0.049	"
Thoti	South Asia	Thoti	28	0.000	Donnelly et al. (2010)

2N* Sample size in number of chromosomes analyzed

Supplementary Table 3. H1', H2', and H2D subhaplotype frequencies in populations from different continents, sample sizes, and references.

**Population**	**Region**	**2N***	**H1´**	**H2´**	**H2D**	**Reference**
Gipuzkoa	Europe	180	0.506	0.006	0.488	Present study
Navarra	Europe	210	0.689	0.033	0.278	"
Valencia	Europe	182	0.742	0.022	0.236	"
Basque Country (residents)	Europe	46	0.717	0.022	0.261	Alfonso-Sánchez et al. (2018)
Adygei	Europe	68	0.794	0.118	0.088	Steinberg et al. (2012)
British	Europe	180	0.755	0.006	0.239	"

Finnish	Europe	200	0.890	0.000	0.110	"
French	Europe	90	0.804	0.018	0.179	"
French Basque	Europe	70	0.729	0.021	0.250	"
North Italian	Europe	30	0.682	0.045	0.273	"
Orcadian	Europe	44	0.733	0.000	0.267	"
Russian	Europe	90	0.900	0.000	0.100	"
Sardinian	Europe	80	0.714	0.054	0.232	"
Spain	Europe	212	0.750	0.000	0.250	"
Tuscany Italian	Europe	200	0.645	0.040	0.315	"
Tuscany Italian	Europe	214	0.636	0.056	0.308	1000G
Bedouin	Middle East	138	0.767	0.022	0.211	Steinberg et al. (2012)
Druze	Middle East	120	0.732	0.024	0.244	"
Palestinian	Middle East	138	0.750	0.022	0.228	"
Balochi	South Asia	88	0.917	0.021	0.063	"
Bengalí	South Asia	170	0.953	0.006	0.041	1000G
Brahui	South Asia	94	0.940	0.040	0.020	Steinberg et al. (2012)
Burusho	South Asia	94	0.940	0.000	0.060	"
Gujarati	South Asia	210	0.910	0.010	0.081	1000G
Hazara	South Asia	86	0.977	0.023	0.000	Steinberg et al. (2012)
Kalash	South Asia	84	0.913	0.000	0.087	"
Makrani	South Asia	86	0.860	0.060	0.080	"
Pathan	South Asia	80	0.909	0.000	0.091	"
Punjabi	South Asia	192	0.901	0.031	0.068	1000G
Sindhi	South Asia	88	0.917	0.021	0.063	Steinberg et al. (2012)
Tamil	South Asia	206	0.971	0.005	0.024	1000G
Telugu	South Asia	206	0.951	0.010	0.039	"
Uygur	Central Asia	40	1.000	0.000	0.000	Steinberg et al. (2012)

Cambodians	East Asia	40	1.000	0.000	0.000	"
Dai	East Asia	40	1.000	0.000	0.000	"
Daur	East Asia	36	1.000	0.000	0.000	"
Han	East Asia	174	0.989	0.000	0.011	"
Han Bejing	East Asia	194	1.000	0.000	0.000	"
Han South	East Asia	204	0.995	0.000	0.005	"
Hezhen	East Asia	32	1.000	0.000	0.000	"
Japanese	East Asia	196	0.995	0.000	0.005	"
Japanese	East Asia	112	1.000	0.000	0.000	"
Kinh	East Asia	202	1.000	0.000	0.000	1000G
Lahu	East Asia	32	1.000	0.000	0.000	Steinberg et al. (2012)
Miaozu	East Asia	40	1.000	0.000	0.000	"
Mongola	East Asia	38	0.950	0.000	0.050	"
Naxi	East Asia	32	1.000	0.000	0.000	"
Oroqen	East Asia	36	1.000	0.000	0.000	"
She	East Asia	40	1.000	0.000	0.000	"
Tu	East Asia	40	1.000	0.000	0.000	"
Tujia	East Asia	40	1.000	0.000	0.000	"
Xibo	East Asia	36	1.000	0.000	0.000	"
Yakut	East Asia	94	0.979	0.000	0.021	"
Yizu	East Asia	40	1.000	0.000	0.000	"
Bakola	Africa	38	0.947	0.053	0.000	"
Bantu-northeast	Africa	22	1.000	0.000	0.000	"
Bantu-south	Africa	16	1.000	0.000	0.000	"
Beja	Africa	36	0.861	0.111	0.028	"
Biaka	Africa	46	0.935	0.043	0.022	"
Boni	Africa	36	0.921	0.000	0.079	"

Borana	Africa	38	0.974	0.000	0.026	"
Bulala	Africa	30	1.000	0.000	0.000	"
Datog	Africa	36	0.917	0.056	0.028	"
Esan	Africa	198	1.000	0.000	0.000	1000G
Fulani	Africa	38	1.000	0.000	0.000	Steinberg et al, (2012)
Gambian	Africa	226	0.987	0.009	0.004	1000G
Hadza	Africa	72	0.972	0.028	0.000	Steinberg et al, (2012)
Iraqw	Africa	36	0.972	0.000	0.028	"
Lemande	Africa	38	1.000	0.000	0.000	"
Luhya	Africa	202	1.000	0.000	0.000	"
Luo	Africa	38	1.000	0.000	0.000	"
Maasai	Africa	286	0.927	0.024	0.049	"
Mandenka	Africa	44	0.977	0.000	0.023	"
Mandera	Africa	36	1.000	0.000	0.000	"
Mbuti	Africa	26	0.923	0.077	0.000	"
Mende	Africa	170	0.994	0.006	0.000	1000G
San	Africa	80	0.975	0.025	0.000	Steinberg et al, (2012)
Sandawe	Africa	92	0.913	0.054	0.033	"
Sengwer	Africa	36	0.917	0.083	0.000	"
Yoruba	Africa	214	1.000	0.000	0.000	"

2N* Sample size in number of chromosomes analyzed

References for Supplementary Tables

1000G (https://www.internationalgenome.org/)

Alfonso-Sánchez, M. A., Espinosa, I., Gómez-Pérez, L., Poveda, A., Rebato, E., Peña, J. A. 2018. Tau haplotypes support the Asian ancestry of the Roma population settled in the Basque Country. Heredity, 120(2), 91-99.

ALFRED (https://alfred.med.yale.edu/alfred/) (Cheung, K. H., Miller, P. L., Kidd, J. R., Kidd, K. K., Osier, M. V., Pakstis, A. J. 1999. ALFRED: a Web-accessible allele frequency database. In Biocomputing 2000, pp. 639-650)

Alkaraki AK, Abuelezz, AI, Khabour, OF, Peña, JA, Alfonso-Sánchez, MA, Altaany, Z, 2021. MAPT haplotypes in Jordan: evidence on the Middle East as a melting-pot predating Neolithic migration. Annals of Human Biology. DOI: 10.1080/03014460.2021.1983018.

Donnelly, M. P., Paschou, P., Grigorenko, E., et al. 2010. The distribution and most recent common ancestor of the 17q21 inversion in humans. The American Journal of Human Genetics, 86(2), 161-171.

Evans, W., Fung, H. C., Steele, J. et al. 2004. The tau H2 haplotype is almost exclusively Caucasian in origin. Neuroscience letters, 369(3), 183-185.

Ghidoni, R., Signorini, S., Barbiero, L., et al. 2006. The H2 MAPT haplotype is associated with familial frontotemporal dementia. Neurobiology of disease, 22(2), 357-362.

Jakobsson, M., Scholz, S. W., Scheet, P., et al. 2008. Genotype, haplotype and copy-number variation in worldwide human populations. Nature, 451(7181), 998-1003.

Skipper, L., Wilkes, K., Toft, M., et al. 2004. Linkage disequilibrium and association of MAPT H1 in Parkinson disease. The American Journal of Human Genetics, 75(4), 669-677.

Stefansson, H., Helgason, A., Thorleifsson, G., et al. 2005. A common inversion under selection in Europeans. Nature genetics, 37(2), 129-137.

Steinberg, K. M., Antonacci, F., Sudmant, P. H., et al. 2012. Structural diversity and African origin of the 17q21. 31 inversion polymorphism. Nature genetics, 44(8), 872-880.

Winkler, S., König, I. R., Lohmann-Hedrich, K., Vieregge, P., Kostic, V., Klein, C. 2007. Role of ethnicity on the association of MAPT H1 haplotypes and subhaplotypes in Parkinson's disease. European journal of human genetics, 15(11), 1163-1168.
